# Factors leading to falls in transfemoral prosthesis users: a case series of sound-side stumble recovery responses

**DOI:** 10.1186/s12984-022-01070-y

**Published:** 2022-09-23

**Authors:** Maura E. Eveld, Shane T. King, Karl E. Zelik, Michael Goldfarb

**Affiliations:** 1 Department of Mechanical Engineering, Vanderbilt University, Nashville, TN USA; 2 Department of Biomedical Engineering, Vanderbilt University, Nashville, TN USA; 3 Department of Physical Medicine and Rehabilitation, Vanderbilt University, Nashville, TN USA; 4 Department of Electrical Engineering, Vanderbilt University, Nashville, TN USA

**Keywords:** Stumble recovery, Trip, Transfemoral prosthesis, Fall prevention

## Abstract

**Background:**

Transfemoral prosthesis users’ high fall rate is related to increased injury risk, medical costs, and fear of falling. Better understanding how stumble conditions (e.g., participant age, prosthesis type, side tripped, and swing phase of perturbation) affect transfemoral prosthesis users could provide insight into response deficiencies and inform fall prevention interventions.

**Methods:**

Six unilateral transfemoral prosthesis users experienced obstacle perturbations to their sound limb in early, mid, and late swing phase. Fall outcome, recovery strategy, and kinematics of each response were recorded to characterize (1) recoveries versus falls for transfemoral prosthesis users and (2) prosthesis user recoveries versus healthy adult recoveries.

**Results:**

Out of 26 stumbles, 15 resulted in falls with five of six transfemoral prosthesis users falling at least once. By contrast, in a previously published study of seven healthy adults comprising 214 stumbles using the same experimental apparatus, no participants fell. The two oldest prosthesis users fell after every stumble, stumbles in mid swing resulted in the most falls, and prosthesis type was not related to strategy/fall outcomes. Prosthesis users who recovered used the elevating strategy in early swing, lowering strategy in late swing, and elevating or lowering/delayed lowering with hopping in mid swing, but exhibited increased contralateral (prosthetic-side) thigh abduction and trunk flexion relative to healthy controls. Falls occurred if the tripped (sound) limb did not reach ample thigh/knee flexion to sufficiently clear the obstacle in the elevating step, or if the prosthetic limb did not facilitate a successful step response after the initial sound-side elevating or lowering step. Such responses generally led to smaller step lengths, less anterior foot positioning, and more forward trunk flexion/flexion velocity in the resulting foot-strikes.

**Conclusions:**

Introducing training (e.g., muscle strength or task-specific motor skill) and/or modifying assistive devices (e.g., lower-limb prostheses or exoskeletons) may improve responses for transfemoral prosthesis users. Specifically, training or exoskeleton assistance could help facilitate sufficient thigh/knee flexion for elevating; training or prosthesis assistance could provide support-limb counteracting torques to aid in elevating; and training or prosthesis assistance could help initiate and safely complete prosthetic swing.

**Supplementary Information:**

The online version contains supplementary material available at 10.1186/s12984-022-01070-y.

## Background

The transfemoral prosthesis user population experiences a substantially high fall rate and accompanying risk of injury, medical costs, and psychosocial effects. In a study of 17 transfemoral prosthesis users who reported falls after a four-week period, an average of 1.25 falls (averaging microprocessor and non-microprocessor knee results) were reported per person [1]. In a similar study examining 19 participants over a 60-day period, an average of 2 falls were reported per person [2]. Interestingly, a recent study reported the healthy adult population’s fall rate to be just 0.37 falls per person per year, though the authors note this work involved a different study design and population size [3]. Furthermore, more than 60% of transfemoral prosthesis users reported falling at least once in the past year [4–6].

With higher fall risk comes increased injury incidence and medical costs. In studies of fall-related injury of lower-limb prosthesis users (transfemoral and transtibial), approximately half of the prosthesis users who fell reported an injury that required medical care [4, 5]. Mundel et al. 2017 [6] reported that the six-month costs of falls resulting in hospitalization for transfemoral prosthesis users are similar to those reported in the elderly population.

Aside from physical and financial consequences, lower-limb prosthesis users experience psychosocial effects of this increased fall prevalence. Forty-seven percent of transfemoral prosthesis users reported a fear of falling [4], and 60% of lower-limb prosthesis users reported that falls affected their daily lives with respect to work, leisure, and confidence [7]. In a focus-group study of lower-limb prosthesis users, participants reported that falls trigger emotions of embarrassment, loss of confidence, fear, and depression, and that they limit participation in certain activities due to risk of falling [8].

In order to develop interventions (e.g., prostheses, training) to decrease fall likelihood, and thus alleviate the physical, monetary, and psychosocial burdens resulting from such falls, it is important to understand both the mechanisms that help prevent falls in healthy populations as well as the deficiencies in recoveries of transfemoral prosthesis users. While responses to trips/stumbles (i.e., obstacle perturbations to the foot in swing phase) in healthy adults have been well characterized, a comparable extent of research for the transfemoral prosthesis user population is lacking.

Three primary responses have been identified and characterized as recovery strategies to swing-phase perturbations (i.e., stumbles or trips) for healthy adults: the elevating, lowering, and delayed lowering strategies [9, 10]. Research has highlighted the kinematics of the tripped limb for each recovery strategy [10–14], the reflexes involved in each recovery strategy [9, 10, 15–18], the role of the support (contralateral) limb during elevating strategies [19–21], and the role of arm movements in successfully recovering from stumbles [22, 23].

For healthy adults, such responses have been found to differ as a function of various factors, including (1) swing phase at which the perturbation occurs and (2) age of the participants. Regarding (1), recovery strategy selected depends on the dynamic state of the human body at the perturbation [24], factors that vary depending on when in swing phase the perturbation occurs. Regarding (2), older adults fall more often, which has been attributed to delayed and decreased muscle activation in the response [16, 19, 25, 26].

While these factors of (1) swing phase and (2) age likely also affect transfemoral prosthesis users’ stumble recovery responses, they may be additionally influenced by factors that are unique to their population, including: (3) knee prosthesis type, and (4) side tripped (i.e., sound limb versus prosthetic limb). Regarding (3), typically prescribed prosthetic knees have various joint designs and control schemes, aspects that affect their behavior after perturbations that are not under the participant’s direct control. A recent study highlighted different rates of falls for wearers of different prosthetic knee models [27]. Regarding (4), it has been shown that both the tripped (ipsilateral) limb and contralateral limb play crucial roles in recovery (one as recovery limb and the other as support limb, depending on strategy) for healthy adults [19–21]; thus, depending on whether the tripped limb is the sound or the prosthetic limb, responses may differ for transfemoral prosthesis users (unlike healthy populations with two identical biological limbs).

Relatively little research has been published examining how factors (1)–(4) affect stumble responses for transfemoral prosthesis users. In a retrospective study of lower-limb prosthesis users, walking was the most commonly reported activity at the time of a fall. Fifty-four percent of falls were attributed to a disruption of the prosthesis user’s base of support (as opposed to a disruption to their center of mass), and 22% were due to tripping specifically [28]. However, further details of how prosthesis users fell, such as which foot was disrupted (i.e., sound versus prosthetic side), and what strategy was attempted following the perturbation, have not been documented in retrospective studies. Thus, while retrospective studies provide important information regarding fall prevalence and effects of falling, it is also crucial to study the real-time nature of stumbles in order to better understand the factors that contribute to falling and hopefully provide insight for mitigation or prevention.

To date, two studies have introduced stumble perturbations to both limbs of transfemoral prosthesis users and report varying results related to factors (1)–(4) [29, 30]. Both studies reported prosthesis user-specific recovery strategies of hopping or skipping after the lowering response. In addition, Crenshaw et al. [29] reported that two out of three sound-side stumbles resulted in a fall (compared to one out of four prosthetic-side stumbles), substantiating the need to study stumbles to the sound limb. They specifically called on future studies to clarify the relationship between stumble features and recovery success. Shirota et al. [30] did not report any falls. Participants in this study were allowed to use handrails, and the rope-blocking perturbation setup did not entail a physical obstacle to clear after stumbling, both factors that may have affected the recovery outcome. However, their results did substantiate Pijnappel et al.’s work that the role of the support limb is critical to recovery [19, 20, 21], further motivating a more comprehensive investigation of sound-side stumble recovery, in which the prosthetic limb is the support limb for elevating strategies.

In order to build upon these prior investigations, and specifically to better understand how different stumble conditions (i.e., participant age, swing phase of perturbation, prosthesis type, and side tripped) affect responses, six transfemoral prosthesis users were recruited to undergo a series of stumble perturbations to both their sound and prosthetic limbs occurring in early, mid, and late swing phase. These experiments elicited substantially different responses depending on which limb was tripped (i.e., sound versus prosthetic), which warranted separate analyses and intervention suggestions. This paper presents the results from tripping the sound limb. Due to limitations in paper length, the results from tripping the prosthetic limb will be presented in a subsequent paper.

Therefore the objective of this work is to examine the responses of transfemoral prosthesis users to sound-side stumble perturbations in order to gain insight into what contributes to falls or facilitates recovery for this population. The primary aim is to characterize their responses by reporting fall outcomes, recovery strategies, and relevant kinematics; the secondary aim is to further analyze these responses to investigate what differentiates recoveries versus falls for transfemoral prosthesis users, as well as what differentiates prosthesis user recoveries from healthy adult recoveries. The intent of this analysis is to provide insights into the deficiencies of transfemoral prosthesis user stumble recovery to better inform fall prevention interventions.

## Methods

### Experimental protocol

Six unilateral transfemoral prosthesis users were recruited for the stumble recovery experiment. Participant details are tabulated in Table 1. All participants wore their prescribed passive prostheses. The prosthetic knee models are noted in Table 1, and the ankles were all energy storage-and-return type prostheses.

**Table 1:** Participant information

Participant	Age	Sex	Prosthetic side	Etiology	Years of prosthesis use	Prescribed prosthesis
HC	25	Male	N/A	N/A	N/A	N/A
P1	62	Male	Right	Trauma	49	Ottobock C-Leg
P2	42	Male	Left	Trauma	14	Ottobock 3R80
P3	28	Female	Right	Congenital	27	Ottobock C-Leg
P4	32	Male	Left	Trauma	4	Blatchford KX06
P5	50	Male	Left	Infection	5	Ottobock C-Leg
P6	30	Male	Left	Trauma	12	Ottobock C-Leg

**Fig. 1:**
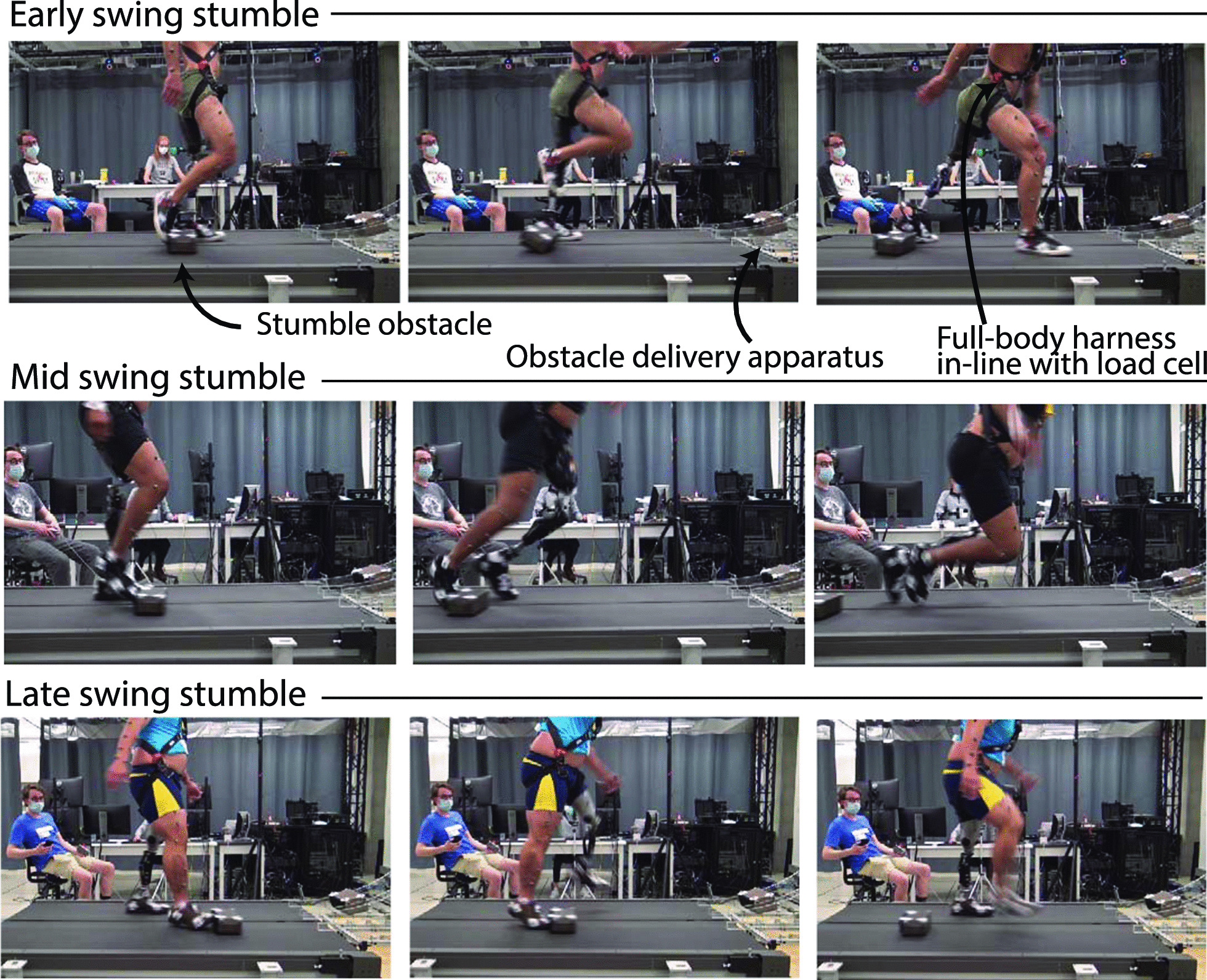
Experimental setup for stumble recovery experiments. Perturbations in early swing (top), mid swing (middle), and late swing (bottom) are pictured here. The stumble perturbation system and experimental protocol are detailed in [14]. Video clips of each stumble trial are included in the Additional files 1, 2, 3

Participants walked on a treadmill at 0.8 m/s and were introduced to a series of obstacle perturbations, targeted to occur in early, mid, and late swing phase (<40%, 40–60%, >60% swing, respectively) for each limb (i.e., prosthetic side and sound side). Specifically, a 35-lb steel block (obstacle) was positioned on a ramp apparatus in front of the treadmill and held in place by an electromagnet; when cued from an onboard targeting algorithm, the electromagnet switches off, releasing the obstacle down the ramp and onto the treadmill belt such that it contacts the participant’s foot at the experimenter-defined percentage of swing phase. The number of steps prior to perturbation, limb stumbled, and swing phase targeted were randomized. Randomization of trials, apparatus design and targeting algorithm, sensory occlusion techniques, and a distraction task were used to ensure the obstacle was unperceived by the participants prior to perturbation and to limit expectation of the stumble. The stumble perturbation system and experimental protocol employed are described in detail and validated experimentally in [14]. Figure 1 depicts the experimental setup with video frames from representative early, mid, and late swing phase stumbles.

The goal was to capture the response to one early, one mid, and one late swing stumble for each limb for each participant. Recall that for this work sound-side stumble responses are presented, while prosthetic-side stumble responses will be reported separately. Depending on the participant’s comfort level, and due to the taxing nature of the protocol, the number of perturbations per session was capped and as such this protocol was completed in either one or two sessions (one session for P2, P3, and P5; two sessions for P1, P4, and P6). Note that the handrails were removed from the treadmill so they could not be used during recovery (in order to mimic a real-life stumble scenario, in which handrails are rarely available), but participants wore a full-body harness to prevent contact with the treadmill in the event of a fall.

Each participant was given the same instructions prior to the stumble trials, namely: walk on the treadmill, perform serial sevens (counting backwards by seven) out loud and monitor visual feedback to stay centered on the treadmill; when the perturbation occurs, try to recover; the treadmill will be stopped to complete the trial when either (1) the participant falls into the harness or (2) the participant recovers and has returned to steady-state walking (qualitatively determined by the experimenters). Participants were given ample time to practice walking on the treadmill without perturbations prior to the stumble trials. All experimental protocols were approved by the Vanderbilt Institutional Review Board, and all participants gave their written informed consent.

### Data collection and processing

Ground reaction forces (GRFs) were recorded under each foot at a sampling rate of 1 kHz via a lateral split-belt, force-instrumented treadmill (Bertec, Columbus, USA). Full-body kinematic data were collected via synchronized infrared motion capture (Vicon, Oxford, GBR) at a sampling rate of 200 Hz. Passive reflective markers were placed bilaterally over the lower limbs at the anterior/posterior superior iliac spines, medial/lateral femoral epicondyles and malleoli, posterior/medial/lateral calcaneus, 1st and 5th metatarsals, and navicular bone; the arms at the acromion, medial/lateral humeral epicondyles, medial/lateral styloid processes; and the torso at the left and right clavicle, sternum, and C7. Clusters of four markers were placed bilaterally on the thigh, shank, upper arm, and forearm. For the prosthetic-side lower limb, markers were placed on the estimated medial/lateral joint center of the prosthetic knee (i.e., femoral epicondyles) and estimated medial/lateral malleoli and foot anatomical landmarks (i.e., calcaneus, metatarsals, navicular bone) of the prosthetic foot. GRF and motion capture data were filtered with a zero-phase, 3rd order, low-pass Butterworth filter with a cut-off frequency of 15 and 6 Hz, respectively. Inverse dynamics were computed using Visual3D (C-Motion, Germantown, USA) to estimate joint-level kinematics and kinetics for each trial.

### Outcome metrics

**Fig. 2:**
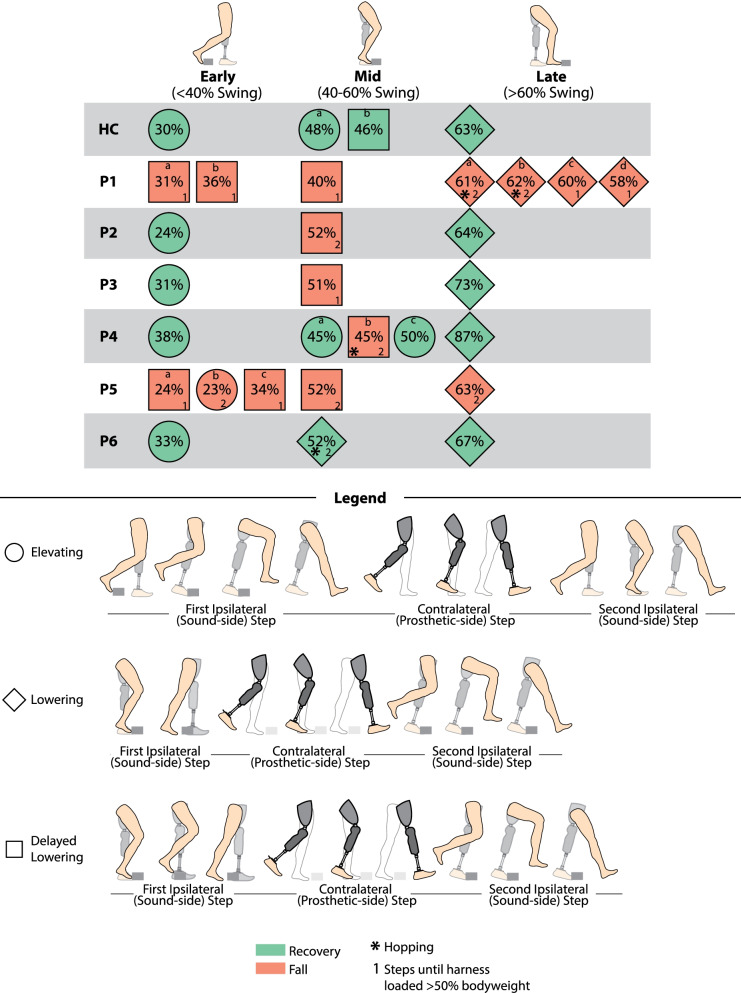
Summary of outcomes for each stumble for each participant. Fall versus recovery, recovery strategy attempted, swing percentage of perturbation, and number of steps prior to a fall (i.e., loading the harness with >50% bodyweight) are provided for each stumble. Video clips of each stumble trial are included in the Additional files 1, 2, 3. If the participant experienced more than one perturbation in a particular bin of swing phase, it is identified by a lower-case letter which is used in subsequent figures and Additional files 1, 2, 3

Several outcome metrics were computed to characterize each stumble response in order to address the primary aim. First, the swing percentage at which the perturbation occurred was estimated as the time from the preceding toe-off event to the instant of perturbation, relative to the average swing time from 20 strides during the same trial. The perturbation was determined as the instant at which the foot contacted the obstacle, which was identified via a transient peak in the anterior-posterior (AP) GRF measured by the treadmill, as in [14]. Second, each trial was labeled as a fall or a recovery based on the force measured by a load cell in-line with the full-body harness. Specifically, if the load cell measured greater than 50% of the participant’s bodyweight, the trial was defined as a fall; otherwise, it was defined as a recovery, similar to [29]. Finally, the recovery strategy used after each perturbation was reported as one of the three previously characterized recovery strategies from healthy adult stumble studies: elevating, lowering, or delayed lowering. Strategies were determined based on the trajectory of the swing foot after perturbation (as in [13, 14, 24]): in the elevating strategy, the foot lifts up and over the obstacle after contact with the obstacle; in the lowering strategy, the foot lowers to the ground behind the obstacle after contact with the obstacle; in the delayed lowering strategy, the foot initially elevates (i.e., shows upward motion) before elevation is abandoned and the foot subsequently lowers to the ground without clearing the obstacle. Refer to the legend of Fig. 2 for representative illustrations of each recovery strategy. Previous studies reported prosthesis user-specific strategies in response to perturbations to the sound side: hopping and skipping [29, 30]. Both of these strategies still involve initial tripped limb lowering or delayed lowering, with the hopping or skipping action as a subsequent response. Thus for this work strategies were reported primarily as elevating, lowering, or delayed lowering, and if hopping or skipping was also utilized, this was additionally documented. For each fall, the number of steps from the time of perturbation to harness loading of >50% bodyweight was also recorded.

Several time-series kinematic trajectories were computed for further characterization of each response. Specifically, ipsilateral (tripped/sound-side) and contralateral (prosthetic-side) sagittal-plane thigh, knee, and ankle angle were computed to characterize and compare the lower-limb motion after each perturbation. Frontal-plane prosthetic-side thigh angle was also computed to characterize the thigh abduction involved in the subsequent step after the stumble. Additionally, sagittal-plane trunk angle (deviation from vertical) after the perturbation was computed, as trunk angle deviations have been reported to be indicators of increased likelihood of falling [31, 32]. Finally, as previous studies have reported the role of arm movements in recovery of healthy adults [22, 23], the trajectories of the forearm center of mass (COM) were computed.

Discrete summary metrics were also extracted in order to address the secondary aim (i.e., to further clarify differences in transfemoral prosthesis user recoveries versus falls, and transfemoral prosthesis user recoveries versus healthy control recoveries). First, discrete metrics capturing key lower-limb dynamics during the response were extracted in order to identify lower-limb distinctions between recoveries and falls: peak ipsilateral (sound-side) thigh flexion, peak ipsilateral knee flexion, peak contralateral (prosthetic-side) thigh flexion, peak contralateral knee flexion, and contralateral knee angle at foot-strike. Second, the work of Grabiner et al. has previously identified several metrics that discriminate falls versus recoveries for various populations by capturing the body’s state at each of the response foot-strikes [12, 31–34]. Interventions that improve these variables and decrease fall risk have been subsequently reported [35–40]. Specifically, time to initial foot-strike, as well as step length (computed as difference in AP position of the stepping foot’s COM to the remaining foot’s COM), AP foot position relative to the body’s COM, trunk flexion, and trunk flexion velocity at each of the recovery foot-strikes have been presented. Thus these variables were also computed for this work in order to (1) illustrate the resulting whole-body outcomes of the aforementioned lower-limb kinematic differences, and (2) align this work with previous studies and potential interventions. Finally, peak trunk angle and trunk angular velocity, as well as peak contralateral (prosthetic-side) thigh abduction, were computed for all recoveries.

### Control comparison data

**Fig. 3:**
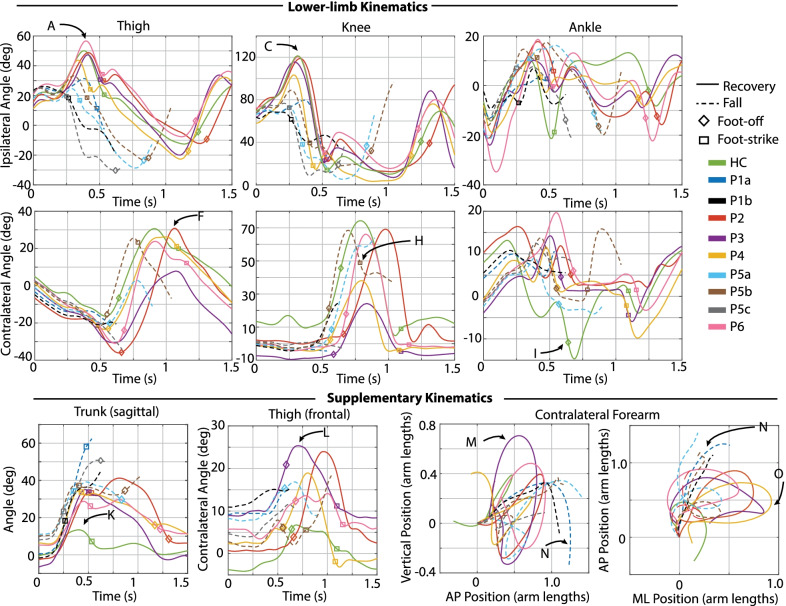
Kinematic characterization for early swing perturbations. Lower-limb kinematics: Sagittal-plane thigh, knee, and ankle angle trajectories for the ipsilateral (tripped/sound, top) and contralateral (support/prosthetic, bottom) limbs after the perturbation. Supplementary kinematics, from left to right: sagittal-plane trunk angle, frontal-plane contralateral thigh angle, contralateral forearm COM trajectory in sagittal and transverse planes. Positive angles indicate sagittal plane joint flexion, frontal plane thigh abduction, and forward trunk angle deviation from vertical. Positive arm positions indicate superior, anterior, and medial to position at perturbation. Arm positions are normalized from the position at perturbation, so trajectories begin at position (0, 0). Lower-limb and trunk trajectories are plotted from the instant of perturbation (Time 0) to either 1.5 s after perturbation (recoveries) or until the participant loaded the harness with >50% bodyweight (falls). Arm trajectories are plotted from the instant of perturbation to either one second after perturbation (recoveries) or until the participant loaded the harness with >50% bodyweight (falls)

**Fig. 4:**
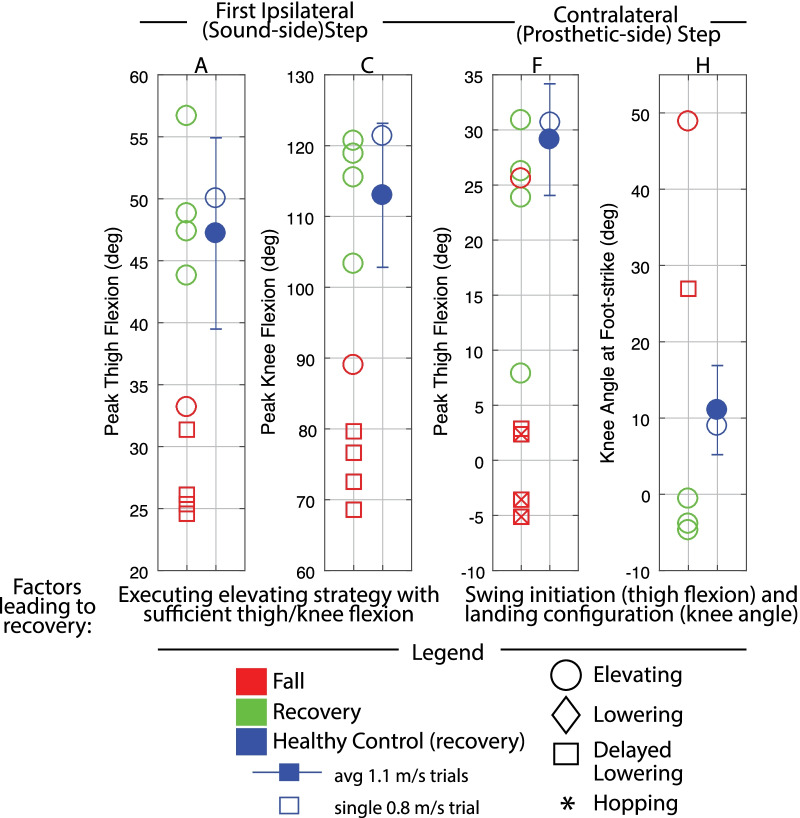
Early swing discrete summary metrics: Lower-limb dynamics. These plots highlight the differences in lower-limb motion between transfemoral prosthesis user falls versus recoveries, with healthy control recovery data included as a reference. Peak thigh and knee flexion in the ipsilateral (tripped/sound-side) step were calculated from perturbation to first ipsilateral foot-strike. Peak thigh flexion in contralateral (prosthetic-side) step was calculated from first ipsilateral foot-strike to first contralateral foot-strike or fall, whichever index occurred first. An “x” in a marker indicates the prosthesis user did not foot-off prior to falling. Capital letters above each plot correspond to letters that are marked in Fig.  3 for reference

**Fig. 5:**
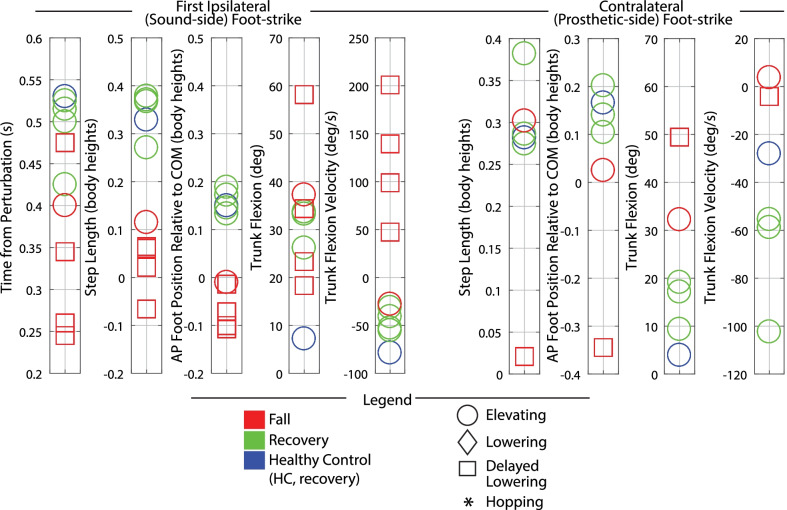
Early swing discrete summary metrics: Foot-strike states. These plots capture the body’s state at each foot-strike after the perturbation, highlighting the differences in falls vs. recoveries for transfemoral prosthesis users. Each metric was computed at the indicated foot-strike. Positive values indicate anterior position, forward trunk flexion, and forward trunk flexion velocity

**Fig. 6:**
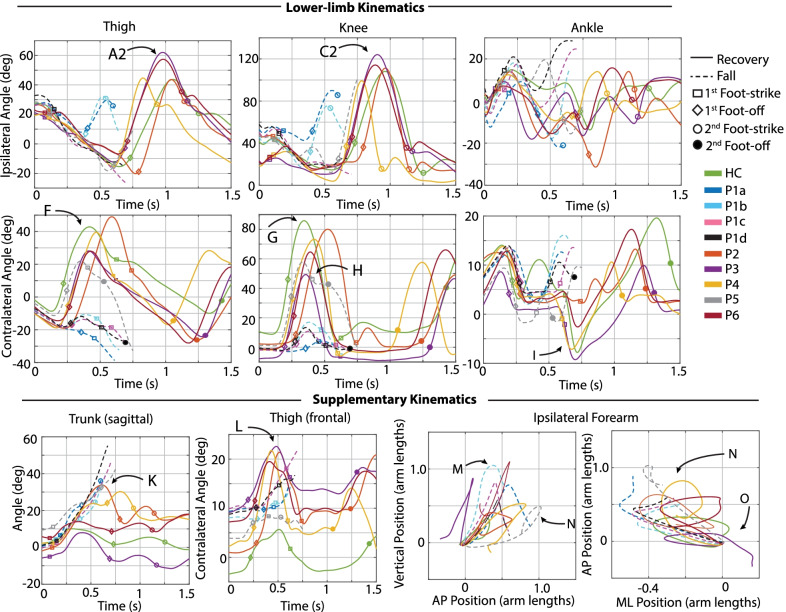
Kinematic characterization for late swing perturbations. Lower-limb kinematics: Sagittal-plane thigh, knee, and ankle angle trajectories for the ipsilateral (tripped/sound, top) and contralateral (recovery/prosthetic, bottom) limbs after the perturbation. Supplementary kinematics, from left to right: sagittal-plane trunk angle, frontal-plane contralateral thigh angle, ipsilateral forearm COM trajectory in sagittal and transverse planes. Positive angles indicate sagittal plane joint flexion, frontal plane thigh abduction, and forward trunk angle deviation from vertical. Positive arm positions indicate superior, anterior, and medial to position at perturbation. Arm positions are normalized from the position at perturbation, so trajectories begin at position (0, 0). Lower-limb and trunk trajectories are plotted from the instant of perturbation (Time 0) to either 1.5 s after perturbation (recoveries) or until the participant loaded the harness with >50% bodyweight (falls). Arm trajectories are plotted from the instant of perturbation to either one second after perturbation (recoveries) or until the participant loaded the harness with >50% bodyweight (falls)

**Fig. 7:**
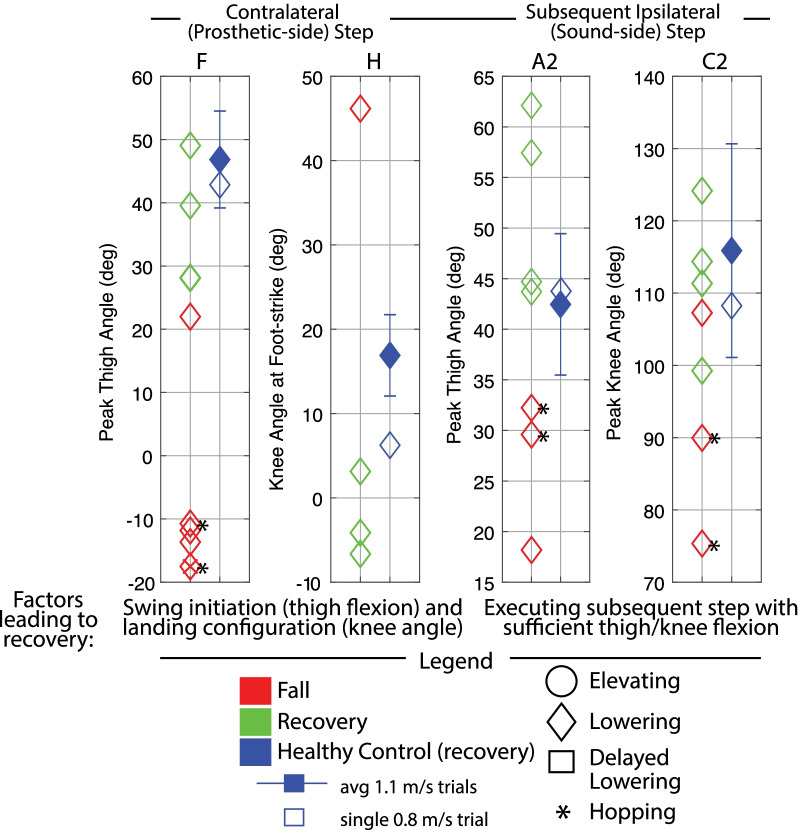
Late swing discrete summary metrics: Lower-limb dynamics. These plots highlight the differences in lower-limb motion between transfemoral prosthesis user falls versus recoveries, with healthy control recovery data included as a reference. Peak thigh flexion in contralateral (prosthetic-side) step was calculated from first ipsilateral foot-strike to first contralateral foot-strike or fall, whichever index occurred first. An “x” in a marker indicates the prosthesis user did not foot-off prior to falling. Peak thigh and knee angle in subsequent ipsilateral (sound-side) step were calculated from ipsilateral foot-off after lowering to foot-strike. Metrics are only plotted if they occurred before the participant loaded the harness with >50% bodyweight (fall). Capital letters above each plot correspond to letters that are marked in Fig.  6 for reference

**Fig. 8:**
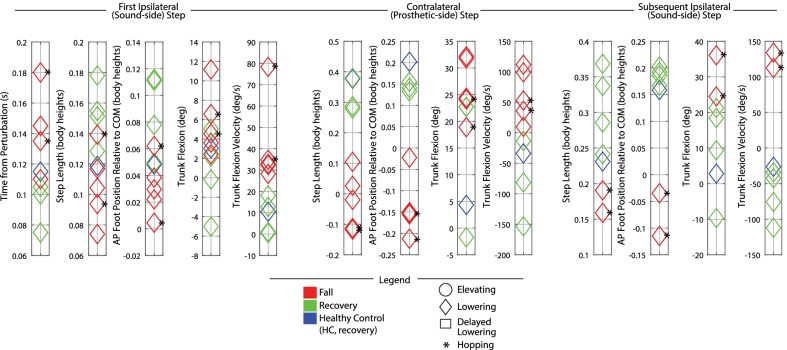
Late swing discrete summary metrics: Foot-strike states. These plots capture the body’s state at each foot-strike after the perturbation, highlighting the differences in falls vs. recoveries for transfemoral prosthesis users. Each metric was computed at the indicated foot-strike. Positive values indicate anterior position, forward trunk flexion, and forward trunk flexion velocity

**Fig. 9:**
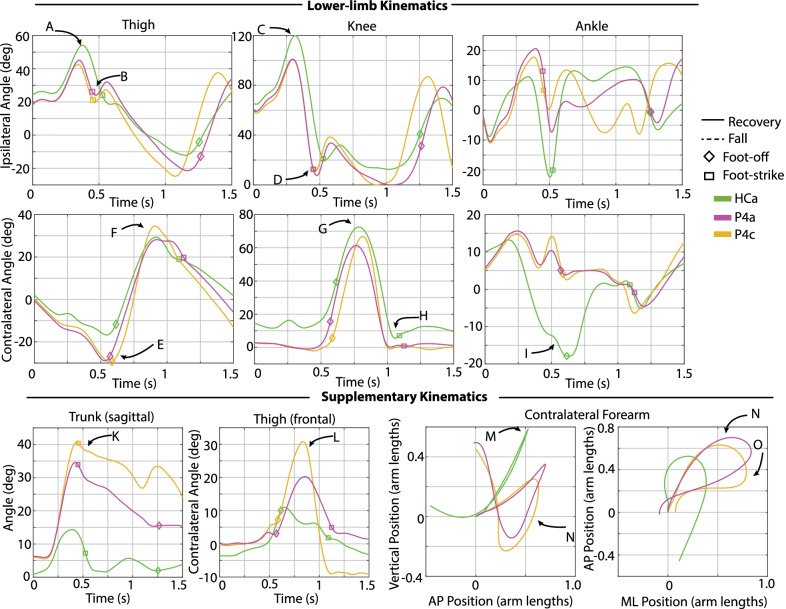
Kinematic characterization for elevating strategies after mid swing perturbations. Lower-limb kinematics: Sagittal-plane thigh, knee, and ankle angle trajectories for the ipsilateral (tripped/sound, top) and contralateral (support/prosthetic, bottom) limbs after the perturbation. Supplementary kinematics, from left to right: sagittal-plane trunk angle, frontal-plane contralateral thigh angle, contralateral forearm COM trajectory in sagittal and transverse planes. Positive angles indicate sagittal plane joint flexion, frontal plane thigh abduction, and forward trunk angle deviation from vertical. Positive arm positions indicate superior, anterior, and medial to position at perturbation. Arm positions are normalized from the position at perturbation, so trajectories begin at position (0, 0). Lower-limb and trunk trajectories are plotted from the instant of perturbation (Time 0) to either 1.5 s after perturbation (recoveries) or until the participant loaded the harness with >50% bodyweight (falls). Arm trajectories are plotted from the instant of perturbation to either one second after perturbation (recoveries) or until the participant loaded the harness with >50% bodyweight (falls)

**Fig. 10:**
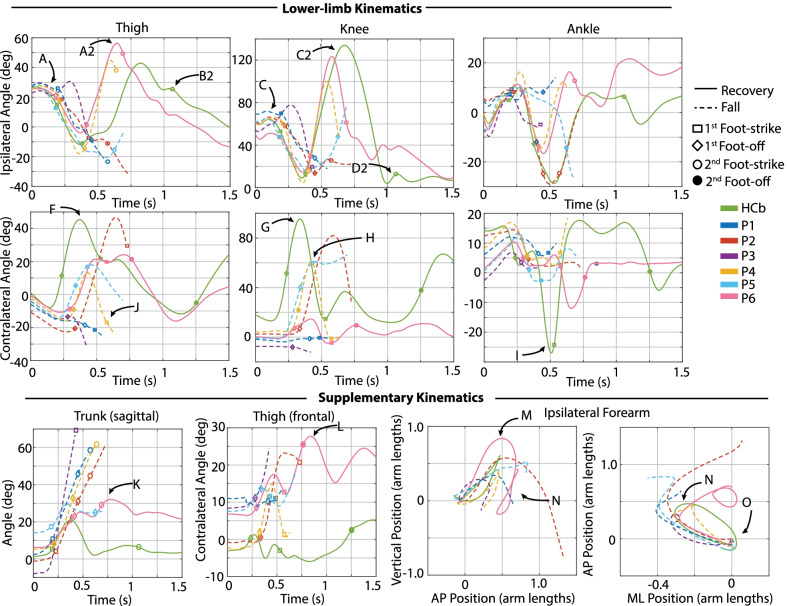
Kinematic characterization for lowering/delayed lowering strategies after mid swing perturbations. Lower-limb kinematics: Sagittal-plane thigh, knee, and ankle angle trajectories for the ipsilateral (tripped/sound, top) and contralateral (recovery/prosthetic, bottom) limbs after the perturbation. Supplementary kinematics, from left to right: sagittal-plane trunk angle, frontal-plane contralateral thigh angle, ipsilateral forearm COM trajectory in sagittal and transverse planes. Positive angles indicate sagittal plane joint flexion, frontal plane thigh abduction, and forward trunk angle deviation from vertical. Positive arm positions indicate superior, anterior, and medial to position at perturbation. Arm positions are normalized from the position at perturbation, so trajectories begin at position (0, 0). Lower-limb and trunk trajectories are plotted from the instant of perturbation (Time 0) to either 1.5 s after perturbation (recoveries) or until the participant loaded the harness with >50% bodyweight (falls). Arm trajectories are plotted from the instant of perturbation to either one second after perturbation (recoveries) or until the participant loaded the harness with >50% bodyweight (falls)

**Fig. 11:**
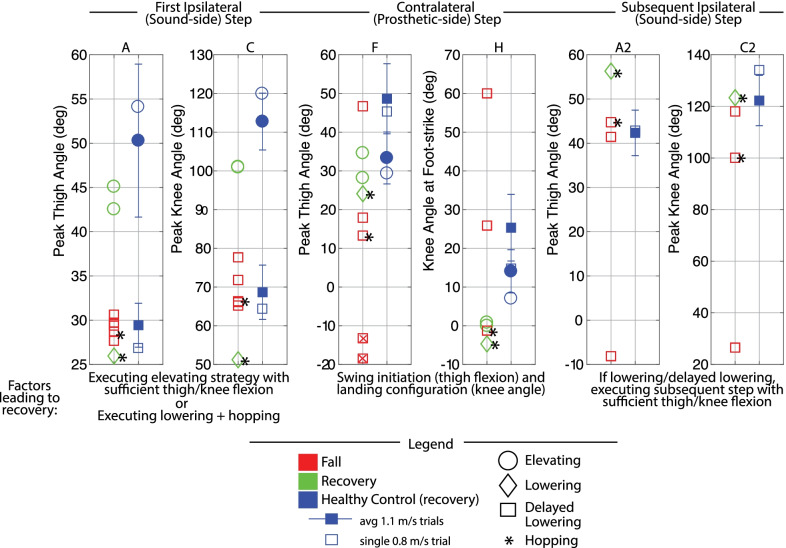
Mid swing discrete metrics: Lower-limb dynamics. These plots highlight the differences in lower-limb motion between transfemoral prosthesis user falls versus recoveries, with healthy control recovery data included as a reference. Peak thigh and knee flexion in the ipsilateral (tripped/sound-side) step were calculated from perturbation to first ipsilateral foot-strike. Peak thigh flexion in contralateral (prosthetic-side) step was calculated from first ipsilateral foot-strike to first contralateral foot-strike or fall, whichever index occurred first. An “x” in a marker indicates the prosthesis user did not foot-off prior to falling. Peak thigh and knee angle in subsequent ipsilateral (sound-side) step were calculated from ipsilateral foot-off after lowering to foot-strike. Metrics are only plotted if they occurred before the participant loaded the harness with >50% bodyweight (fall). Capital letters above each plot correspond to letters that are marked in Fig.  9 and  10 for reference

**Fig. 12:**
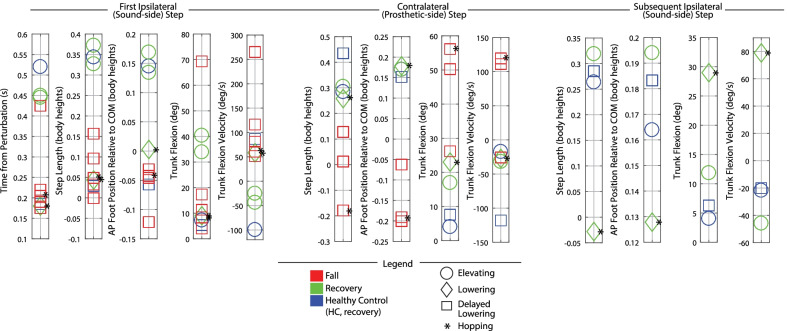
Mid swing discrete metrics: Foot-strike states. These plots capture the body’s state at each foot-strike after the perturbation, highlighting the differences in falls vs. recoveries for transfemoral prosthesis users. Each metric was computed at the indicated foot-strike. Positive values indicate anterior position, forward trunk flexion, and forward trunk flexion velocity

**Fig. 13:**
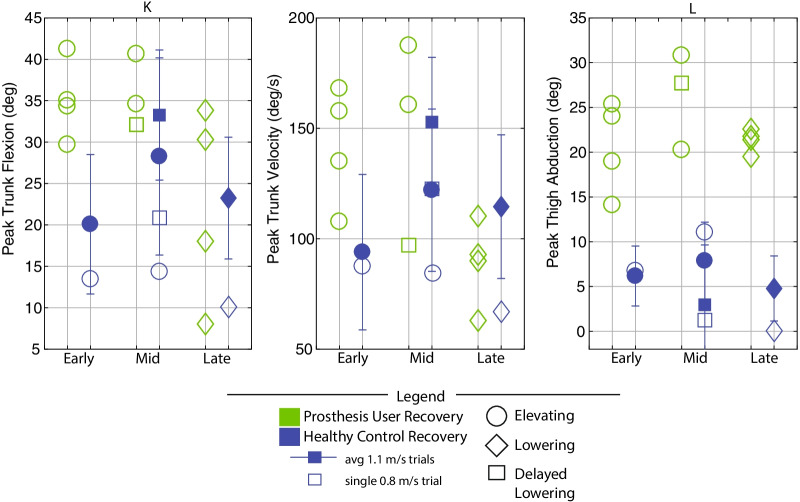
Discrete summary metrics comparing transfemoral prosthesis user recoveries versus healthy control recoveries. Peak trunk flexion and flexion velocity were calculated as the peak value from perturbation to 1.5 seconds after the perturbation. Peak prosthetic-side thigh abduction was calculated as peak frontal-plane thigh angle from contralateral foot-off to contralateral foot-strike. Capital letters above each plot correspond to letters that are marked in Figs.  3,  6,  9, and  10 for reference

Data from a previous healthy adult stumble study [14, 24] were used for comparison to the responses of transfemoral prosthesis users. Seven healthy adults underwent a similar protocol using the same experimental apparatus, in which each participant experienced approximately 28 obstacle perturbations while walking at 1.1 m/s. These data collectively represent 86 elevating strategies in early swing, 40 elevating strategies in mid swing, 24 delayed lowering strategies in mid swing, and 38 lowering/delayed lowering strategies in late swing. Although not exactly speed-matched with the experiments described here, these data nonetheless provide insights regarding which metrics correspond to successful recovery responses, and thus better illustrate prospective deficiencies in responses that result in a fall. Another experiment was also conducted in which a single healthy adult participant repeated the protocol at a walking speed of 0.8 m/s [24]. The healthy participants were wearing the same marker set, and motion capture and GRF data were processed identically to the transfemoral prosthesis user dataset. Data from the seven healthy participants, along with (separately identified) data from the single speed-matched healthy control participant (hereafter referred to as HC, details reported in Table 1), is provided as a reference in Figs. 4, 7, 11, and 13 below. Data from a representative trial from HC are included in Figs. 3, 5, 6, 8, 9, 10, and 12.

## Results

Fifteen out of the 26 stumbles to the sound side of the transfemoral prosthesis users resulted in falls. This is in stark contrast to the healthy participants, in which no falls occurred out of 214 total stumbles.

Of the 26 transfemoral prosthesis user stumble responses, seven were elevating, nine were delayed lowering, and 10 were lowering. Four hopping strategies were used after lowering or delayed lowering strategies. No skipping strategies were observed. Five out of six participants fell at least once. Two participants fell after all perturbations, and three participants only fell after perturbations in mid swing. The breakdown of fall outcomes and strategies used for each participant are reported in Fig. 2.

Figures 3, 6, 9, and 10 provide time-series kinematic trajectories of the lower limbs, trunk, and arms after each individual perturbation for early, late, and mid swing stumbles. Note that Figs. 9 and 10 separate the mid swing responses into elevating strategies and lowering/delayed lowering strategies, respectively, for visual clarity. To supplement these figures, textual descriptions of the kinematics of each response are provided, organized by falls and recoveries for early, late, and mid swing stumbles. Videos of each stumble response are also available in Additional files 1, 2, 3.

Discrete summary metrics from Figs. 3, 6, 9, and 10 are plotted separately in Figs. 4,  7, and  11 to highlight lower-limb kinematic differences in falls versus recoveries among transfemoral prosthesis users. Figures 5, 8, and 12 plot the resulting foot-strike configuration states to further distinguish falls and recoveries. Finally, Fig. 13 plots peak trunk and thigh abduction metrics to highlight differences in transfemoral prosthesis user recoveries from healthy control recoveries.

### Early swing

#### Recoveries

All four recoveries from early swing perturbations were accomplished by the elevating strategy, in which the tripped limb lifted up and over the obstacle in the same step, landing anterior to the obstacle. Refer to the legend of Fig. 2 for a representative illustration of this response. These responses (P2, P3, P4 and P6) are characterized by ipsilateral thigh flexion (peak 44–57 deg) and knee flexion (peak 104–121 deg) during the elevating step, trajectories comparable to the HC elevating step (Fig. 3A and C). However, transfemoral prosthesis users exhibited 7–23 deg more contralateral (prosthetic-side) thigh abduction in the following step and substantially more peak trunk flexion and flexion velocity than the HC response (Fig. 3L and K). Also of note, HC used 11 deg contralateral ankle plantarflexion during the elevating step, a position not available to the prosthesis users due to their passive prosthetic ankles (Fig. 3I). Regarding arm movements, transfemoral prosthesis users exhibited similar contralateral forearm trajectories, characterized by initial deviation superior, anterior, and medial to the position at perturbation before returning to that position. HC employed a similar trajectory but with less overall displacement (Fig. 3M–O).

#### Falls

Two of the six participants fell consistently in response to early swing perturbations (Fig. 2). Of note, these two participants fell after all perturbations to their sound limb. These early swing falls occurred after one elevating strategy and four delayed lowering strategies. During the elevating strategy, P5b just cleared the obstacle using substantially less thigh and knee flexion than non-fallers (Fig. 3A and C) and landing well before HC, P2, P3, and P6 (as indicated with ipsilateral foot-strike in Fig. 3 and Time from Perturbation metric in Fig.  5). In his subsequent contralateral (prosthetic-side) step, he did not reach full knee extension and landed with a flexed knee (Fig. 3H). In the remaining early swing stumbles, P1 and P5 initially elevated but ultimately abandoned elevating and lowered without clearing the obstacle (i.e., delayed lowering). P5a fell during the swing phase of his attempted contralateral (prosthetic-side) step that he did not complete, as evidenced by substantially less contralateral thigh flexion (Fig. 3F). In the remaining falls, P5c and P1a,b could not initiate the contralateral step, as evidenced by the lack of contralateral (prosthetic-side) thigh flexion/knee flexion after the delayed lowering step (Fig. 3F). Transfemoral prosthesis user arm trajectories were characterized by initial deviation superior and anterior from the initial position but never returned to that position before loading the harness with >50% bodyweight (Fig. 3M–O).

#### Discrete summary metrics: transfemoral prosthesis user recoveries versus falls in early swing

*Lower-limb dynamics* As shown in Fig. 4, transfemoral prosthesis users who recovered from early swing stumbles exhibited higher thigh and knee flexion in the first ipsilateral (sound-side) step after perturbation. These kinematics indicate employing a successful elevating strategy to clear the obstacle; note this is in contrast to the substantially less thigh/knee flexion used in the single elevating and four delayed lowering strategies that resulted in falls. Additionally, the prosthesis users who recovered used more peak thigh flexion in the contralateral (prosthetic-side) step and landed with an extended knee. These kinematics reflect successfully initiating swing phase on the prosthetic limb and landing in a safe configuration, as opposed to a lack of thigh flexion and/or knee flexion at foot-strike which resulted in falls. Note that data from HC (single healthy participant tripped at 0.8 m/s) and the seven-participant average of early swing responses resemble kinematics used by prosthesis users who recovered.

*Foot-strike states* As shown in Fig. 5, compared to the elevating strategy fall, elevating strategy recoveries involved a longer time from perturbation to first foot-strike, as well as a greater step length and more anterior foot position relative to body COM at the first (sound-side) and second (prosthetic-side) foot-strikes. At the second foot-strike, elevating recoveries also exhibited less trunk flexion and more negative (backward) trunk flexion velocity compared to the elevating fall. Lowering strategies always resulted in falls for early swing stumbles, which were characterized by a positive (forward) trunk flexion velocity at first foot-strike. Only one lowering strategy completed the next ground contact (prosthetic-side, recovery step), which involved more trunk flexion, less negative trunk angular velocity, a shorter step length, and foot position more posterior to the body COM relative to recoveries.

### Late swing

#### Recoveries

All late swing recoveries were accomplished by the lowering strategy, in which the tripped limb was immediately lowered to the ground, terminating that step and initiating a step with the contralateral (prosthetic) limb, followed by another ipsilateral (sound-side) step to clear the obstacle. Refer to the legend of Fig. 2 for a representative illustration of this response. Transfemoral prosthesis users exhibited 5–35 deg less knee flexion and 20-23 deg more thigh abduction in contralateral step (Fig. 6G and L) compared to HC. Three transfemoral prosthesis users (P2, P4, P6) exhibited more trunk flexion after the perturbation than HC (Fig. 6K). Transfemoral prosthesis users exhibited similar ipsilateral arm trajectories, characterized by initial deviation superior, anterior, and lateral to the original position before returning to that position. HC employed a similar trajectory but with less overall displacement (Fig. 6M–O).

#### Falls

Two of the six participants fell consistently in response to late swing perturbations (Fig. 2). Of note, these two participants fell after all perturbations to their sound limb. The five falls (P1a, b, c, d and P5) were all attempted lowering strategies. P1a, b lowered the tripped (sound) limb to the ground, then attempted to hop off the same limb (hopping) rather than initiate a contralateral step, as evidenced by the ipsilateral thigh and knee flexion after foot-strike and lack of contralateral thigh flexion after the perturbation (Fig. 6A2, C2, F). After P1’s remaining two late swing perturbations (P1c,d), P1 fell immediately after lowering the tripped limb to the ground and no other recovery steps were attempted, as shown by lack of initiation of ipsilateral thigh flexion or contralateral thigh and knee flexion (Fig. 6A2, F, G). P5 lowered the tripped limb to the ground and initiated a contralateral step, as evidenced by contralateral thigh flexion, but landed much sooner than the non-fallers’ (P2, P3, P4, P6) contralateral step with a more flexed knee, as shown in the timing and knee flexion at contralateral foot-strike (Fig. 6H).

#### Discrete summary metrics: transfemoral prosthesis user recoveries versus falls in late swing

*Lower-limb dynamics* As shown in Fig. 7, transfemoral prosthesis users who recovered exhibited substantially higher contralateral (prosthetic-side) thigh flexion and landed with a more extended knee. These kinematics reflect successfully initiating swing phase on the prosthetic limb and landing in a safe configuration, as opposed to a lack of thigh flexion and/or knee flexion at foot-strike which resulted in falls. Additionally, prosthesis users who recovered reached higher thigh and knee flexion in the subsequent ipsilateral step. These kinematics reflect successfully clearing the obstacle, as opposed to lack of thigh/knee flexion exhibited by those who fell after lowering or lowering with hopping. Note that data from HC and the seven-participant average of late swing responses resemble kinematics used by prosthesis users who recovered.

*Foot-strike states* As shown in Fig. 8, compared to lowering strategy falls, lowering strategy recoveries generally involved less time from perturbation to first foot-strike, greater step length, more anterior foot position relative to COM, less trunk flexion, and more negative (backward) trunk flexion velocity at first foot-strike (initial sound-limb loading), the next foot-strike (prosthetic-side recovery step), and the subsequent sound-side foot-strike.

### Mid swing

#### Recoveries

Recoveries in mid swing were accomplished by two elevating strategies (P4a, c) and one lowering strategy with hopping (P6a). As shown in Fig. 9, during the elevating strategies (P4) the tripped limb lifted up and over the obstacle in the same step, landing anterior to the obstacle, as HCa did. However, HC exhibited substantially higher peak thigh and knee flexion during the elevating step (10 deg and 20 deg more, respectively) and landed later than P4 (Fig. 9A–D). During the subsequent contralateral (prosthetic-side) step, P4 exhibited substantially more thigh extension in the sagittal plane (Fig. 9E) and abduction in the frontal plane (Fig. 9L), less knee flexion (Fig. 9G), less ankle plantarflexion (Fig. 9I), and more trunk flexion (23–30 deg, Fig. 9K) than HC. Contralateral arm motion followed similar trajectories to that of the early swing elevating strategies of transfemoral prosthesis users, with HC again using less overall arm displacement (Fig. 9M–O).

As shown in Fig. 10, P6 recovered using a lowering with hopping strategy; specifically, upon contacting the obstacle he immediately terminated his step and lowered behind the obstacle, but subsequently hopped off of his ipsilateral (sound) limb. During the hop the contralateral (prosthetic-side) limb swung laterally (substantial thigh abduction without knee flexion) to facilitate the following step and landed just before the second ipsilateral step (versus a $\sim$500 ms difference between foot-strikes with HCb, indicated by foot-strike marker in Fig. 10). HCb exhibited substantially more thigh and knee flexion and ankle plantarflexion in the contralateral step (Fig. 10F, G, I), then landed with a more extended thigh and knee in the subsequent ipsilateral step (Fig. 10B2, D2). HC also exhibited 13 deg less peak trunk flexion and 15 deg less thigh abduction after the perturbation (Fig. 10K, L).

#### Falls

Five of the six participants fell during mid swing perturbations. P4 attempted a delayed lowering with hopping strategy, in which upon contacting the obstacle he initially elevated but ultimately terminated his step and lowered behind the obstacle, then subsequently hopped but experienced substantial trunk flexion just as he landed and loaded the harness with >50% bodyweight. P4 landed with his prosthetic-side thigh substantially more extended than P6, as evidenced by contralateral thigh angle at foot-strike (Fig. 10J). P2 attempted a delayed lowering strategy, in which he initially elevated upon contacting the obstacle, but ultimately lowered behind the obstacle, followed by a contralateral step, but did not succeed in landing, experiencing substantial trunk flexion before loading the harness with >50% bodyweight. P5 also attempted a delayed lowering strategy and initiated a contralateral (prosthetic-side step), but landed with substantial knee flexion, as evidenced in contralateral knee angle at foot-strike (Fig. 10H), which buckled the knee and led to a fall. Finally, P1 and P3 attempted delayed lowering strategies but were unable to initiate a contralateral (prosthetic-side) step after the lowering step before falling, as evidenced by lack of contralateral thigh flexion (Fig. 10F). Fallers’ ipsilateral arm motions were characterized by arm motion superior, anterior, and lateral to the position at perturbation (Fig. 10M–O).

#### Discrete summary metrics: transfemoral prosthesis user recoveries versus falls in mid swing

*Lower-limb dynamics* Transfemoral prosthesis users who recovered after mid swing stumbles either elevated or lowered with hopping. Thus kinematics that differentiate falls versus recoveries (Fig. 11) reflect the description/trends noted for early swing elevating strategies from Fig. 4 (sufficient thigh/knee flexion in first ipsilateral step, and sufficient thigh flexion and knee extension at foot-strike in contralateral step), or late swing lowering strategies from Fig. 7 (sufficient thigh flexion and knee extension at foot-strike in contralateral step after lowering, and sufficient thigh/knee flexion in next ipsilateral step to clear obstacle). Recall that for the successful lowering strategy in mid swing, a hopping strategy was also employed. Note that data from HC (single healthy participant tripped at 0.8 m/s) and the seven-participant average of early swing responses resemble kinematics used by prosthesis users who recovered.

*Foot-strike states* The mid swing elevating strategy recoveries involved step times, step lengths, foot positions relative to COM, trunk flexion, and trunk flexion velocities comparable to early swing elevating strategies. Compared to mid swing lowering strategy falls, the mid swing delayed lowering strategy recovery involved a more anterior foot position relative to COM at first sound-side foot-strike, as well as a greater step length, more anterior foot position relative to COM, and less trunk flexion in the next contralateral (prosthetic-side) foot-strike.

### Discrete summary metrics: transfemoral prosthesis user recoveries versus healthy control recoveries in early, mid, and late swing

As shown in Fig. 13, early and mid swing perturbations induced approximately double the effect on transfemoral prosthesis users (versus healthy participants) in terms of peak trunk flexion and flexion velocity. The majority of prosthesis user late swing stumble responses also involved more peak trunk flexion and flexion velocity than HC (single 0.8 m/s trial). Additionally, for early, mid, and late swing stumbles, prosthesis users employed substantially more thigh abduction in the contralateral (prosthetic-side) step during recovery compared to healthy control data (7–19 deg for early, 10–20 deg for mid, and 15–19 deg for late).

Note that relative to HC, the prosthesis users who recovered with an elevating strategy used a comparable step length and foot positioning at first foot-strike, but landed with more trunk flexion and more forward trunk flexion velocity (Figs. 5 and 12). During lowering/delayed lowering strategies, HC took a larger contralateral step with more anterior foot positioning relative to COM than the majority of prosthesis user recoveries (Figs. 8 and 12).

## Discussion

Five of the six transfemoral prosthesis users fell at least once in this study, while none of the seven healthy control participants fell in [14, 24]. These results substantiate the high fall prevalence relative to healthy adults reported in retrospective studies [1–4, 7, 41, 42], highlighting that stumbles to the sound limb should not be overlooked when considering interventions for fall prevention.

The following subsections provide an in-depth discussion of results with respect to the stumble conditions of (1) swing phase, (2) participant age, and (3) prosthesis type, as well as a commentary on arm motion. Finally, based on these observations, several interventions for improving recovery and mitigating falls for the transfemoral prosthesis user population are considered.

### Considering swing phase

#### Early

Transfemoral prosthesis users who recovered in early swing (P2, P3, P4, P6) used an elevating strategy, as HC did. This was accomplished via substantial sound-side thigh and knee flexion to complete the elevating step, and a successful initiation (thigh flexion) and completion (extended knee angle) of the next prosthetic-side step (Fig. 4); this lower-limb motion allowed prosthesis users to land with a greater step length, more anterior foot position relative to COM, less trunk flexion, and more negative trunk velocity at each of the recovery foot-strikes (Fig. 5), aligning with the work of Grabiner et al. which has shown that an improvement in these metrics (i.e., trending in the direction described) indicates a decreased fall risk [31–34, 43]. However, transfemoral prosthesis users who recovered exhibited more support limb (prosthetic-side) thigh abduction in the next step and increased trunk flexion and flexion velocity compared to healthy control data (Fig. 13). Thigh abduction is likely a compensation mechanism in order to facilitate swing phase during the non-cyclic activity of stumble recovery (see Considering Prosthesis Type subsection). Increased trunk flexion has been reported as an indicator of increased fall likelihood [31, 32] and may be due to lack of moment generation of the passive prosthetic limb joints (See Considering Age, Commentary on Arm Motion, and Interventions for Consideration subsections). Likewise, contralateral ankle plantarflexion employed by HC during the elevating step (not observed for prosthesis users) may have helped reduce forward angular momentum and thus limit trunk flexion during recovery [21, 44, 45] (see Considering Age and Interventions for Consideration subsections).

Transfemoral prosthesis users who fell in early swing (P1, P5) either inadequately performed the elevating strategy or attempted a delayed lowering strategy but could not successfully initiate a contralateral (prosthetic-side) step. In the seven-participant healthy adult study, all 86 stumbles that occurred before 40% swing phase resulted in elevating strategies (i.e., none abandoned elevating to perform the delayed lowering strategy) [14, 24]. In this study, the transfemoral prosthesis users who used the delayed lowering strategy (i.e., abandoned elevating) exhibited substantially high forward trunk flexion velocity at initial sound-side foot-strike, and only one could complete the second prosthetic-side step, suggesting that employing the delayed lowering strategy in early swing may increase the body’s forward angular momentum enough to contribute to falls. This increase in trunk flexion and forward trunk flexion velocity at recovery foot-strikes aligns with the work of Grabiner et al. [12, 31–34, 40, 43], which has used these metrics as fall indicators for other populations.

These early swing findings are inconsistent with the only other study of sound-side, early swing perturbations with transfemoral prosthesis users, in which majority lowering strategies and no falls were reported [30]. This may be due to the ability of participants in that study to use the handrails and/or to the lack of a physical obstacle to clear in the rope-blocking apparatus.

Collectively these results suggest that properly performing the elevating strategy is key for recovering from early swing stumbles.

#### Late

Transfemoral prosthesis users who recovered in late swing (P2, P3, P4, P6) used a lowering strategy, as HC did. This was accomplished via substantial thigh flexion and a safe landing configuration for the prosthetic-side step as well as substantial sound-side thigh and knee flexion in the subsequent step to clear the obstacle (Fig. 4); this lower-limb motion allowed users to land with a larger step length, more anterior foot position relative to COM, and more negative trunk flexion velocity at the prosthetic-side foot-strike and subsequent sound-side foot-strike (Fig. 8), again aligning with trends seen in [31–40, 43, 46].

However, lowering strategy recoveries involved more trunk flexion and more prosthetic-side thigh abduction compared to healthy controls (Fig. 13) likely in part due to deficiencies in stance release of their prescribed knee prosthesis (see Considering Prosthesis Type subsection).

The transfemoral prosthesis users who fell in late swing (P1, P5) all attempted a lowering strategy but could not initiate and/or complete the next (prosthetic-side) step. This was evidenced by the lack of prosthetic-side thigh flexion or a flexed knee at prosthetic-side foot-strike (Fig. 4), which contributed to the smaller step length, posterior foot position relative to COM, and more forward trunk flexion velocity at prosthetic-side foot-strike (Fig. 8), metrics that agree with previously reported fall indicators. Note that these lowering strategy falls were also characterized by a longer time to initial loading (first sound-side foot-strike), which is consistent with findings of [31].

These late swing findings are mostly consistent with Shirota et al. [30], who also observed lowering strategies, as well as with Crenshaw et al. [29], who observed one elevating (fall), one lowering with hopping (recovery), and one lowering (fall), and suggested that lack of knee control contributed to the difficulty of executing the recovery step with the prosthetic limb.

Collectively these results suggest that properly initiating swing after the lowering step, landing in a safe (i.e., extended knee) configuration on the prosthetic limb, and using sufficient thigh/knee flexion to clear the obstacle in the subsequent sound-side step are key for recovering from late swing stumbles.

#### Mid

Transfemoral prosthesis users who recovered in mid swing (P4, P6) used an elevating or lowering with hopping strategy. The trends observed in early swing elevating strategies and late swing lowering strategies extend to mid swing regarding lower-limb dynamics (Fig. 11) and foot-strike states (Fig. 12).

These mid swing findings are mostly consistent with Shirota et al. [30] in terms of strategy selection. However, while Shirota et al. observed no falls, this mid swing region presented the highest likelihood of falls across participants (i.e., five of six fell) in the present study, suggesting that this region may warrant particular attention when considering interventions. The lowering with hopping response was qualitatively similar to the hopping strategy discussed in previous works [29, 30]. Crenshaw et al. 2013 [29] notes that this strategy may be difficult for individuals with plantarflexion weakness; indeed, in this study only one participant successfully recovered in this way.

Collectively these results suggest that either properly performing the elevating strategy or properly initiating prosthetic-side swing after the lowering step (for a more normative, without hopping, lowering/delayed lowering strategy) is key for recovering from mid swing stumbles.

### Considering age

It is notable that the two oldest participants in this case series (P1 and P5) were the only two participants to fall after every perturbation. These results are consistent with several studies of healthy (non-prosthesis user) older and younger adults, in which the older adults fell more [19, 41] which was attributed to delayed and/or diminished muscle responses [16, 19]. Pijnappels et al. [25] highlights that older adults have problems meeting the requirements for adequate balance recovery, since muscle strength, reaction time, and coordination decline with age. Specifically, Pijnappels et al. [19] reported significantly longer rise times of electromyography (EMG) amplitudes of the biceps femoris, gastrocnemius medialis, and soleus muscles in the support limb of the older adults, which reduces rate of force generation. This finding is likely compounded in the present study by the fact that in the case of elevating strategies, the support limb is the prosthetic limb, in which these three muscles have been altered or removed due to amputation/congenital limb difference (discussed more in the Commentary on Arm Motion and Interventions for Consideration subsections). Pijnappels et al. and others conclude that a combination of resistance/strength training and task-specific motor skill training has the potential to improve responses [25, 34], which are discussed in the Interventions for Consideration section.

Pavol et al. 1999 [47] reported in their overground stumble study of older adults that the participants who took more rapid steps had significantly increased likelihood of falling after a trip. Additionally, gait asymmetry metrics have been used to determine and quantify gait pathologies [48]. Thus the cadence and prosthetic-to-sound limb swing time symmetry were computed for each participant. P5 walked with a substantially increased cadence (i.e., more rapid steps, at 115 steps/min) compared to the remaining participants (87–97 steps/min). P1 and P5 also walked with higher swing-time asymmetry (ratio of 1.52 and 1.41, respectively) compared to the remaining participants (1.23–1.31). Therefore, age of participant may be a proxy for other step metrics that indicate higher fall risk.

### Considering prosthesis type

Four of the transfemoral prosthesis user participants wore an Ottobock C-Leg (P1, P3, P5, P6), which is a hydraulic-based microprocessor-controlled knee (MPK). Two participants wore hydraulic non-MPKs: P2 wore an Ottobock 3R80, a single-axis, rotary-hydraulic knee; P4 wore a Blatchford KX06, a four-bar, hydraulic knee. All six are hydraulic knees with stance control function (i.e., high resistance against flexion during stance). Stance resistance against flexion is initiated at heel-strike in the non-MPKs, while it is initiated during swing extension in the C-Leg. The C-Leg and 3R80 are both single-axis knees, while the KX06 employs 4-bar kinematics that increase toe clearance during swing phase and also enhance stability during stance phase.

For early and late swing perturbations, there was no clear trend in prosthesis type contributing to falls versus recoveries. Recall that the two transfemoral prosthesis users who fell were also the oldest participants, which was discussed in the Considering Age subsection. However, the transfemoral prosthesis users who recovered (regardless of prosthesis type) used substantial thigh abduction during the subsequent prosthetic-side step (Fig. 13), likely to help initiate swing at a time when the prescribed device was not in the proper mode. Both non-MPKs and MPKs rely on various sensing thresholds to allow for stance release, as well as the ballistic coordination with the thigh for swing phase motion, both of which are reliable during the cyclic motion of walking; however, these prostheses do not account for the interruption of inertial swing dynamics that occurs during sound-side stumbles.

For mid swing perturbations, the participant who recovered using an elevating strategy wore a non-MPK (P4 with KX06). The participant who recovered using a lowering strategy wore an MPK (P6 with C-Leg). However, it is unclear whether the devices themselves helped facilitate recovery; especially in the case of P6, the C-Leg’s lack of stance release likely led to the hopping response after lowering that required substantial thigh abduction to facilitate swing and involved increased trunk flexion. Thus, the recoveries in mid swing are potentially attributed to the ability of the prosthesis user rather than the prosthesis itself. For the delayed lowering responses (all falls), the non-MPK users (P2 and P4) were able to initiate a subsequent step with their prosthetic side after the lowering step (Fig. 10), while the MPK users could not initiate this subsequent step. Thus, the non-MPKs may have some advantage at more quickly initiating swing; however, their capabilities were not robust enough to successfully complete the step.

There was one instance of prosthetic knee buckling, in which P5 did not take a full prosthetic-side step after his delayed lowering step and landed with the knee substantially flexed (Figs. 10 and 11).

From these observations, for sound-side stumble recovery there does not seem to be a clear advantage of knee prosthesis type. Instead, both demonstrated similar deficits: namely, the inability to initiate swing and/or complete swing during the next step. All participants wore similar energy storage-and-return type prosthetic feet, and all showed similar deficits in terms of lack of active plantarflexion and less range of motion compared to HC. The effect of prosthetic foot model was beyond the scope of this work but could be investigated in future studies.

### Commentary on arm motion

Roos et al. 2008 [22] concluded that arm movements contribute to stumble recovery for healthy adults by both elevating the body’s center of mass and reducing its forward angular momentum, which provide more time for the positioning of the recovery limb. Pijnappels et al. 2010 [23] concluded that arm responses counteracted transverse plane body rotation which helped with recovery foot positioning. For all three strategies, but particularly with elevating, transfemoral prosthesis user recoveries were characterized by substantially more vertical, anterior, and medial/lateral deviation of the forearm from its position at perturbation (prior to returning to that position) as well as increased trunk flexion compared to HC (Figs. 3, 6, 9, 10, and 13). As discussed in the Considering Swing Phase and Interventions for Consideration subsections, Pijnappels et al. [21] conclude that the reactive torques of the support limb of healthy adults enable the necessary push-off reaction, thus reducing the forward angular momentum of the body and providing more time for positioning of the elevating limb. Given that the support limb was the prosthetic limb (i.e., no active ankle or knee power and compromised muscles) for elevating strategies in this study, perhaps the exaggerated arm motion observed among transfemoral prosthesis users is necessary to counter the lack of moment generation of the support limb, allowing for reduced forward angular momentum and/or better foot placement to avoid falling.

Interestingly, the transfemoral prosthesis users who fell after every perturbation were the two oldest participants, and they employed arm motion similar to those found in the older participants of Roos et al. 2008 [22]; described as a more “protective” strategy, these arm movements were characterized by more anterior displacement (a forward reaching-like motion that suggested bracing themselves for an expected fall), rather than the “preventive” strategy employed by younger adults to counteract loss of balance (Figs. 3, 6, 9, and 10).

### Interventions for consideration

#### What causes falls?

In this case series, fall incidence was related to swing percentage (the mid swing stumbles resulted in the most falls) and age (the oldest prosthesis users fell after every stumble), but not with prosthesis type (MPK versus non-MPK), as discussed in the previous discussion subsections.

Falls for this population can be attributed to two main deficiencies in the transfemoral prosthesis users’ responses after a stumble. First, falls occurred if the tripped (sound) limb did not reach ample thigh and knee flexion to sufficiently clear the obstacle in the elevating step (Fig. 4 and  11A and C). The inability to perform the elevating strategy may come from a decrease in strength or reaction time of the tripped (sound) limb (see Considering Age subsection), and/or a lack of counteracting moment generation in the support (prosthetic) limb (see Considering Swing Phase and Commentary on Arm Motion subsections). Recall that even the elevating strategy recoveries involved more trunk flexion, forward trunk flexion velocity, and arm motion than healthy controls, also likely due to these two factors.

Second, falls occurred if the prosthetic limb did not facilitate a successful step response (Fig. 7 and 11F and H) after the initial elevating or lowering sound-side step; specifically, either prosthetic swing was not initiated (as evidenced by lack of thigh flexion before falling) or swing was initiated but the prosthesis landed in an unsafe configuration (i.e., knee flexed). The inability to perform this prosthetic-side step is likely due to their prescribed prostheses’ control scheme that does not decrease flexion resistance until stance-release thresholds are met, as well as its passive behavior that relies on ballistic coordination with the thigh for full swing phase motion, both of which are compromised when the cyclic motion of gait is interrupted during a stumble (see Considering Prosthesis Type subsection). Recall that even the elevating and lowering strategy recoveries involved more thigh abduction in the prosthetic-side step, also likely due to this factor.

Both of these deficiencies in lower-limb dynamics (highlighted in Figs. 4, 7, and 11) overall led to shorter steps, less anterior foot placement relative to COM, and more trunk flexion/flexion velocity at each of the response foot-strikes (highlighted in Figs. 5, 8, and 12) for fallers compared to non-fallers, confirming that these previously reported fall indicator metrics [12, 31–39] translate to the transfemoral prosthesis user population.

These observations suggest that appropriate interventions to decrease fall risk for transfemoral prosthesis users may be to assist in properly performing the elevating strategy and/or to assist in initiating and safely completing the prosthetic-side step. Such interventions could be accomplished with some combination of training and external assistance, discussed below.

#### Training interventions

Strength training and/or task-specific motor skill training may help prosthesis users recover by targeting the two aforementioned deficiencies.

First, strength training targeted at the sound (tripped) limb’s hip and knee flexors may improve success in elevating over the obstacle. (Note one could potentially also accomplish this with a powered exoskeleton on the sound limb.) Training targeted at the prosthetic-side (support limb) hip flexors could also help by providing counteracting torques during the elevating step; however, transfemoral prosthesis users still may lack in the necessary knee or ankle torques due to their passive prostheses. Additionally, training targeted at the prosthetic-side hip could help initiate swing phase; however, the transfemoral prosthesis users who recovered in this study employed substantial thigh abduction to accomplish the step due to prosthesis constraints, likely necessitating external interventions discussed subsequently. Strength training has shown potential for improving responses for fall-prone populations [25]; future work is needed to investigate the feasibility of such training for prosthesis users for sound-side stumbles.

Second, a task-specific training protocol in the form of repeatedly introduced treadmill acceleration disturbances that require a stepping response may also improve recovery outcomes. In particular, the work of Grabiner et al. has shown that this task-specific training can improve the metrics at response foot-strikes linked to increased fall risk (reported in Figs. 5, 8, and 12 [34, 49]), and ultimately reduce fall incidence for some populations [43]. Future work is needed to investigate the feasibility and lasting effect of such training for transfemoral prosthesis users for sound-side stumbles.

#### Prosthesis interventions

Powered prostheses (e.g., [50, 51, 52]) may reduce fall risk by addressing the deficiencies of the typically prescribed passive prosthetic knees in responses to stumbles.

First, a powered prosthesis could improve the sound limb elevating response by providing the necessary counteracting ankle and/or knee torques in the support limb, allowing for a more normative push-off to facilitate the elevating step and reduce the body’s forward angular momentum [19, 30].

Second, a powered prosthesis could presumably sense a stumble and initiate powered swing phase for the subsequent step without requiring ballistic coordination with the thigh, as passive prostheses do. In other words, users could more easily initiate and complete swing phase in the following step, addressing one of the leading causes of falls among study participants. Additionally, powered prostheses can provide robust stance support even if the prosthesis lands with a flexed knee. Some energetically passive knees could provide robust stance support as well, although in general, any prosthesis that does so must implement this as a stumble-specific behavior, since otherwise doing so would interfere with stance knee yielding during slope or stair descent.

Such improvements in the lower-limb deficiencies observed may improve the body’s state at prosthetic-side foot-strike (i.e., larger step length, more anterior foot placement, less trunk flexion, and more negative trunk flexion velocity).

Despite this potential for improvement, to date there have been no prosthetic interventions developed or tested to address sound-side stumbles for transfemoral prosthesis users. Future work is needed to investigate the feasibility of such interventions.

These suggestions may not only reduce falls but also generally improve responses for transfemoral prosthesis users who did not fall. For example, transfemoral prosthesis users still exhibited responses that indicated increased risk of falling (increased trunk flexion/flexion velocity) and compensation mechanisms (increased thigh abduction and arm motion) relative to healthy controls; thus, training and/or assistive device interventions may improve these metrics, ultimately helping to reduce the need to abduct and help control forward angular momentum to reduce these deficiencies.

### Limitations

There are several limitations to this work. First, a sample size of more participants would have improved the confidence of the observations enumerated here. More non-MPK users would have improved the discussion on the effect of prosthesis type. Regardless, given the inherent heterogeneity of the prosthesis user population (i.e., varying ages, activity levels, comorbidities, years of prosthesis use, prosthesis types), a case series characterization such as that presented here is arguably more representative than one in which a singular averaged result is reported [53].

Second, while substantial efforts were made experimentally to ensure the obstacle was not perceived prior to contacting the participant’s foot (see Methods and Results of [14]), the participants did know that they would be stumbled at some point during the trial.

Third, there is potential that requiring a cognitive task during the trials may alter balance outcomes; however, a recent stumble study found that performing Serial Sevens (i.e., the cognitive task chosen in this work) did not alter the participants’ recovery response [54]. Furthermore, in the real world tripping often occurs when individuals are distracted/not paying attention [55, 56], so the task is not entirely unlike real-life situations.

Fourth, rest was not standardized across participants; instead, participants were given the opportunity to rest as much as desired in between each stumble based on their own comfort and energy levels. This rest period was based on personal preference rather than physiological monitoring, so there is potential that fatigue could have affected responses. However, there were no observed trends in fall/recovery outcomes with order of trials (e.g., no evidence that more falls occurred towards the end of a session) across participants.

Finally, the authors note that there are other factors that may have played a role in responses: socket attachment, residual muscle condition, and physical fitness level. Though this type of analysis was beyond the scope of the present work, future studies investigating these factors are encouraged.

## Conclusion

This study presents a case series of sound-side stumble responses to obstacle perturbations in early, mid and late swing phase for six transfemoral prosthesis users of various ages and prosthesis types. Five out of six participants fell at least once—in contrast to a similar study of seven healthy participants in which none fell—highlighting the importance of studying sound-side stumbles and considering appropriate interventions. Stumbles elicited in mid swing resulted in the most falls (five out of six participants fell), suggesting a potential region of focus for intervention design. The two oldest participants were the only two to fall after every stumble, highlighting age-related differences in muscle strength and control and consequently ability to recover. Prosthesis type (specifically MPK versus non-MPK) did not relate to strategy or fall outcomes among the six participants studied; rather, both prosthesis types exhibited similar deficits regarding inability to initiate and/or complete swing phase.

Strategies that resulted in recoveries for transfemoral prosthesis users matched those used by the healthy control participant; namely the elevating in early swing, the lowering in late swing, and either the elevating or the lowering/delayed lowering (with hopping) in mid swing. However, these recoveries still showed kinematic variations from the control participant, characterized by increased contralateral (prosthetic-side) thigh abduction, increased trunk flexion and flexion velocity, and exaggerated arm motions. Thigh abduction is likely a compensation performed to help initiate/complete swing phase on the prosthetic side. Trunk and arm responses are likely affected by the nature of the passive prosthetic support limb, which cannot generate the counteracting torques typically generated by healthy musculature. Those who fell inadequately performed these recovery strategies, evidenced by lower-limb deficiencies in executing the elevating step and/or in initiating and completing the prosthetic-side step, which led to a shorter step length, less anterior foot placement relative to COM, more trunk flexion, and more forward trunk flexion velocity at each foot-strike, metrics that have been previously reported as fall indicators.

These observations suggest that interventions via training (e.g., muscle strength or task-specific motor skill) and/or assistive devices (e.g., lower-limb prostheses or exoskeletons) may address the main response deficiencies that led to falls for transfemoral prosthesis users. Specifically, training or exoskeleton assistance could help facilitate sufficient thigh/knee flexion for the elevating strategy; training or prosthesis assistance could provide counteracting torques in the support limb to facilitate elevating; and training or prosthesis assistance could help initiate and safely complete prosthetic swing.

## Supplementary information

**Additional file 1.** A compilation video of each participant's stumble responses in early swing (< 40% swing phase). If a participant experienced more than one perturbation in early swing, it is identified by a lower-case letter which is used in the main text and figures. Refer to Fig. 2 for fall/recovery outcome, strategy, and swing percentage of each stumble.

**Additional file 2.** A compilation video of each participant's stumble responses in mid swing (40-60% swing phase). If a participant experienced more than one perturbation in mid swing, it is identified by a lower-case letter which is used in the main text and figures. Refer to Fig. 2 for fall/recovery outcome, strategy, and swing percentage of each stumble.

**Additional file 3.** A compilation video of each participant's stumble responses in late swing (> 60% swing phase). If a participant experienced more than one perturbation in late swing, it is identified by a lower-case letter which is used in the main text and figures. Refer to Fig. 2 for fall/recovery outcome, strategy, and swing percentage of each stumble.

## Data Availability

The kinematic datasets used and/or analyzed during the current study are available from the corresponding author on reasonable request.

## Abbreviations

GRF: ground reaction force
AP: anterior-posterior
COM: center of mass
HC: healthy control
P1–P6: Participant 1–Participant 6
EMG: electromyography
